# Whole genome sequencing of ESBL-producing *Escherichia coli* isolated from patients, farm waste and canals in Thailand

**DOI:** 10.1186/s13073-017-0471-8

**Published:** 2017-09-06

**Authors:** Chakkaphan Runcharoen, Kathy E. Raven, Sandra Reuter, Teemu Kallonen, Suporn Paksanont, Jeeranan Thammachote, Suthatip Anun, Beth Blane, Julian Parkhill, Sharon J. Peacock, Narisara Chantratita

**Affiliations:** 1 Department of Microbiology and Immunology, Faculty of Tropical Medicine, Mahidol University, 420/6 Rajvithi Road, Bangkok, 10400 Thailand; 2 Department of Medicine, University of Cambridge, Box 157 Addenbrooke’s Hospital, Hills Road, Cambridge, CB2 0QQ UK; 3 Division of Clinical Microbiology, Medical Technology Department, Bhuddhasothon Hospital, Chachoengsao, 24000 Thailand; 4 The Wellcome Trust Sanger Institute, Wellcome Genome Campus, Hinxton, Cambridge, CB10 1SA UK; 5 London School of Hygiene and Tropical Medicine, London, WC1E 7HT UK

**Keywords:** *Escherichia coli*, ESBL, Genome, Sequence, Phylogeny

## Abstract

**Background:**

Tackling multidrug-resistant *Escherichia coli* requires evidence from One Health studies that capture numerous potential reservoirs in circumscribed geographic areas.

**Methods:**

We conducted a survey of extended β-lactamase (ESBL)-producing *E. coli* isolated from patients, canals and livestock wastewater in eastern Thailand between 2014 and 2015, and analyzed isolates using whole genome sequencing.

**Results:**

The bacterial collection of 149 isolates consisted of 84 isolates from a single hospital and 65 from the hospital sewer, canals and farm wastewater within a 20 km radius. *E. coli* ST131 predominated the clinical collection (28.6%), but was uncommon in the environment. Genome-based comparison of *E. coli* from infected patients and their immediate environment indicated low genetic similarity overall between the two, although three clinical–environmental isolate pairs differed by ≤ 5 single nucleotide polymorphisms. Thai *E. coli* isolates were dispersed throughout a phylogenetic tree containing a global *E. coli* collection. All Thai ESBL-positive *E. coli* isolates were multidrug resistant, including high rates of resistance to tobramycin (77.2%), gentamicin (77.2%), ciprofloxacin (67.8%) and trimethoprim (68.5%). ESBL was encoded by six different CTX-M elements and SHV-12. Three isolates from clinical samples (*n* = 2) or a hospital sewer (*n* = 1) were resistant to the carbapenem drugs (encoded by NDM-1, NDM-5 or GES-5), and three isolates (clinical (*n* = 1) and canal water (*n* = 2)) were resistant to colistin (encoded by *mcr*-*1*); no isolates were resistant to both carbapenems and colistin.

**Conclusions:**

Tackling ESBL-producing *E. coli* in this setting will be challenging based on widespread distribution, but the low prevalence of resistance to carbapenems and colistin suggests that efforts are now required to prevent these from becoming ubiquitous.

**Electronic supplementary material:**

The online version of this article (doi:10.1186/s13073-017-0471-8) contains supplementary material, which is available to authorized users.

## Background

The global spread of multidrug resistant bacteria is a major threat to human health. This includes drug-resistant *Escherichia coli*, a leading cause of bloodstream and urinary tract infections [1]. Extended spectrum β-lactamase (ESBL)-producing *E. coli* are capable of hydrolysing numerous antibiotics, including third-generation cephalosporins [1]. This impacts on patient outcome, since infection with ESBL-producing *E. coli* is associated with higher mortality, longer length of hospital stay and increased costs compared to infection with antibiotic-susceptible *E. coli* [2]. The challenge to successful therapy has further increased following the emergence of multidrug-resistant isolates with acquired resistance to the carbapenem drugs and more recently to colistin, a drug of last resort for multidrug-resistant infections [3, 4]. Transferrable colistin resistance is mediated by *mcr*-*1* or *mcr*-*2* carried on a plasmid, and has been detected in *E. coli* isolated in Europe, Africa and Asia [4, 5].

Tackling multidrug-resistant bacteria requires an understanding of their reservoirs and routes of spread. *E. coli* are normal gut commensals for humans and other animals, including livestock, and can be isolated from food and the environment [6, 7]. Studies are increasingly reporting isolation of multidrug-resistant *E. coli* from livestock, such as chickens, ducks, pigs and cattle in Asia (China, South Korea and Lebanon) and Europe (the UK and the Netherlands), as well as meat and the environment [8–13]. ESBL-producing *E. coli* can also persist in the farm environment for prolonged periods [14], and become concentrated in the surrounding environment through repeated contamination with farm wastewater [11]. Water may also provide a mechanism for the further dissemination of ESBL-producing *E. coli* across more extended distances. This could increase the risk of human acquisition through the consumption of contaminated drinking water [15].

The need to understand the relationship between *E. coli* from different reservoirs through One Health studies is well known, but requires a discriminatory bacterial typing technique. The increasing application of whole genome sequencing brings a level of discrimination and information on relatedness and resistance mechanisms that surpass previous typing methods [16]. This has been used to compare the whole genomes of environmental, commensal and pathogenic *E. coli* to understand their ecology and speciation [17]. Several studies have used genome sequencing to characterize multidrug-resistant *E. coli* with a particular focus on ST131, which has become a dominant clinical clone worldwide [18–20]. The use of whole genome sequencing in countries engaged in intensive livestock farming, which have been proposed to be at high risk for the emergence of drug resistance, is important. One such country is Thailand, which is a major producer and exporter of chickens. A recent study reported that nearly 78% of *E. coli* isolated from pig and broiler carcass samples from slaughterhouses in eastern Thailand were multidrug resistant [21]. In addition, ESBL-producing *E. coli* was highly prevalent in samples from healthy adults (76%), healthy pigs (77%) and broiler chickens (40%), and in water samples collected from farms (33%) and canals in central Thailand (25%) [7]. A limitation of this study was that genotyping was not performed to examine strain relatedness or define the mechanisms of resistance. Here, we describe the findings of a survey of ESBL-producing *E. coli* isolated from patients, canals and livestock wastewater in a defined region of eastern Thailand in which isolates were evaluated using whole genome sequencing.

## Methods

### Study design and bacterial isolates

The bacterial collection consisted of 149 ESBL-positive *E. coli* isolated between 2014 and 2015. Clinical isolates (*n* = 84) were from consecutive positive samples processed by the diagnostic microbiology laboratory at Bhuddhasothorn hospital, Chachoengsao province, eastern Thailand between December 2014 and April 2015. Date of isolation and sample type were recorded, and only one isolate per patient was included. Isolate details are shown in Additional file 1: Table S1. Bacterial isolates were initially identified and susceptibility testing performed using Standard Operating Procedures supplied by the Department of Medical Science, Ministry of Public Heath, Thailand and Clinical and Laboratory Standards Institute (CLSI) guidelines (M100-S24 and M100-S25), respectively. Species was subsequently confirmed using matrix-assisted laser desorption/ionization time-of-flight mass spectrometry (MALDI-TOF MS; Biotyper version 3.1, Bruker Daltonics, Coventry, UK). Antimicrobial susceptibility testing was repeated using the N206 card on the Vitek 2 instrument (bioMérieux, Marcy l’Étoile, France) calibrated against EUCAST breakpoints, and these results used during the analysis. E-test (bioMérieux, Marcy l’Étoile, France) was used when further verification was required.

Environmental isolates (*n* = 65) were obtained through a cross-sectional survey between January 2015 and February 2015 described previously [22]. In brief, wastewater samples were collected from 27 canals and 11 farms within a 20 km radius of Bhuddhasothorn hospital. The farms reared pigs (*n* = 2), chickens (*n* = 6), ducks (*n* = 2) and both chickens and ducks (*n* = 1), where samples were collected from gullies that drained waste from animal housing. The geographical position of each sampling site was recorded using GPSMAP 60CSx (Garmin, Taiwan). A further two wastewater samples were taken from the Bhuddhasothorn hospital wastewater treatment system (one pre-treatment and one post-treatment water sample). Maps of the study region were created using ArcGIS software version 10.3.1.

### Wastewater processing and bacterial identification

Samples were processed by filtration onto membranes as described previously [22], which were incubated on ESBL Brilliance agar (Oxoid, Basingstoke, UK) for 48 h at 35 °C in air. Up to ten colonies suspected to be *E. coli* based on colour were picked and screened for ESBL expression using a phenotypic confirmation test based on CLSI guidelines (M100-S25). Species and antimicrobial susceptibility testing were confirmed using MALDI-TOF MS and Vitek 2, as described above. All bacterial isolates were stored at −80 °C in trypticase soy broth with 20% glycerol.

### Whole genome sequencing and data analysis

DNA extraction, sequencing and assembly of reads were performed as described previously [23]. Sequencing was performed on an Illumina HiSeq2000. Genomes were assembled using Velvet with the improvements described previously [24]. Details of reads, numbers of contigs and N50 are provided in Additional file 2: Table S2. Multilocus sequence types (MLST) were identified from the sequence data using the Achtman scheme and an in-house script (https://github.com/sanger-pathogens/mlst_check). Sequence data have been deposited in the European Nucleotide Archive (ENA; http://www.ebi.ac.uk/ena) under the individual accession numbers given in Additional file 2: Table S2.

The pan-genome was estimated for the 149 study genomes using Roary, with a minimum percentage identity for blastp of 90%, and an alignment created of all core genes (present in 99% of isolates) [25]. Single nucleotide polymorphisms (SNPs) in the core genes were extracted and used to construct a maximum likelihood tree using RAxML with 100 bootstraps and a midpoint root. Additionally, the study genomes were contextualised against a global collection. Sequence data for 514 *E. coli* isolates reported previously [26–28] were downloaded from the ENA and the Wellcome Trust Sanger Institute Pathogen Genomics pipeline and annotated using Prokka. The 514 global genomes were combined with the 149 study genomes and the pan-genome estimated using Roary. SNPs in the core genes were used to construct a maximum likelihood tree as above.

To place the 26 ST131 study isolates into a global context, sequence data for 319 ST131 isolates reported previously [18, 19, 29] were downloaded from the ENA. These, together with the 26 ST131 study genomes, were mapped to *E. coli* NTCC13441 (accession number LT632320-LT632321) using SMALT (http://www.sanger.ac.uk/science/tools/smalt-0). All isolates had greater than 95× coverage. Mobile genetic elements were identified and removed as described previously [30]. Recombination was removed using Gubbins [31]. SNPs were identified and used to construct a maximum likelihood tree. Four isolates from Price et al. [32] and three isolates from Stoesser et al. [33] were filtered out by Gubbins, leaving a total of 312 external and 26 study isolates in the phylogeny. FimH typing was performed by in silico PCR with previously published primers [34] and the resulting products compared to the sequences of known FimH types [35] using blastn. The H30R and H30Rx sub-clones were classified by H30Rx-specific SNPs as described previously [18] using in silico PCR.

### Identification of antimicrobial resistance determinants, virulence factors and plasmids

To detect acquired genes encoding antimicrobial resistance, a manually curated version of the ResFinder database (compiled in 2012) was used [36]. Sequences were compared to this as described previously and genes with > 90% identity match were classified as present. The presence of the *mcr*-*1* and *mcr*-*2* genes was detected using in silico PCR with previously published primers [5, 37]. Sequences of the seven CTX-M genes identified here (CTX-M14-1 (JF701188), CTX-M14-48 (AJ416341), CTX-M15-23 (DQ302097), CTX-M24-8 (EF374096), CTX-M27-1 (EU916273), CTX-M55-2 (GQ456159) and CTX-M65-2 (GQ456158)) were downloaded from the ENA and a phylogeny created using FastTree [38]. Putative virulence genes were collated from the Virulence Factors of Pathogenic Bacteria Database (http://www.mgc.ac.cn/VFs/; *n*  284). The presence of virulence factors was determined using an in-house script (https://github.com/simonrharris/map_resistome) with genes classed as present if there was a match with > 90% length and > 90% identity. Statistical analyses were performed using Fisher’s exact test with a *p* value of 0.05.

## Results

### Isolation of ESBL-producing *E. coli* from patients and the environment

**Fig. 1:**
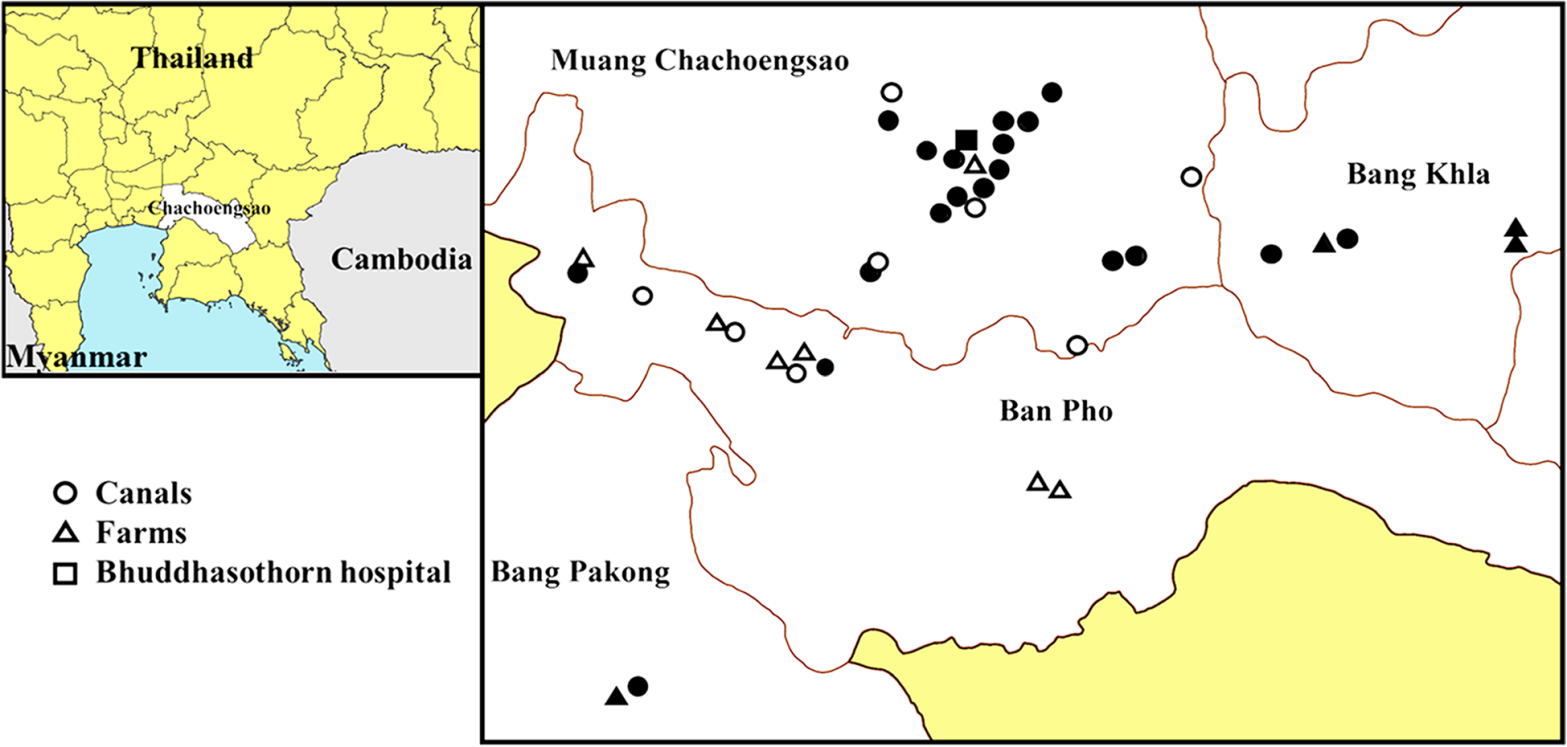
Map of sampling. Geographical location of sampling points. *Left*: map indicating the position of Chachoengsao province in East Thailand (*white area*, Chachoengsao province; *yellow areas*, other Thai provinces). *Right*: map indicating environmental sampling points in individual main districts (amphoe) within Chachoengsao province. *Black symbols*, positive for ESBL-positive *E. coli*; *white symbols*, ESBL-positive *E. coli* not isolated. Map created using ArcGIS software by Esri

A cross-sectional survey of canals and untreated waste from farms and a hospital in Chachoengsao province, Thailand led to the recovery of 65 ESBL-positive *E. coli*. Fifty-six were isolated from canals, three from pre-treated hospital wastewater and six from wastewater from four different farms (Fig. 1). A longitudinal survey of ESBL-positive *E. coli* isolated from clinical samples from the microbiology laboratory at Bhuddhasothorn hospital between December 2014 and April 2015 led to the identification of 84 isolates from blood (21 isolates), urine (39), pus (23) and sputum (1). Sequence types (STs) were identified from the whole genome sequence data. Taken together, the 149 ESBL-positive *E. coli* isolates were assigned to 72 STs, although the clinical isolate collection (84 isolates, 30 STs) was less diverse than the environmental isolate collection (65 isolates, 53 STs). The prevalence of the eight most frequent STs identified in each of the clinical or environmental collections is shown in Additional file 3: Figure S1. ST131 was the predominant ST in the clinical collection (24/84, 28.6%), whilst no single ST predominated in the environmental collection.

### Antibiotic resistance

**Fig. 2:**
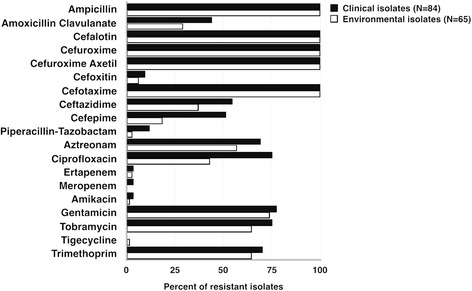
Phenotypic antimicrobial resistance for ESBL-producing *E. coli* from clinical and environmental sources. The percentage of clinical (*n* = 84) and environmental (*n* = 65) isolates resistant to each antibiotic tested

**Fig. 3:**
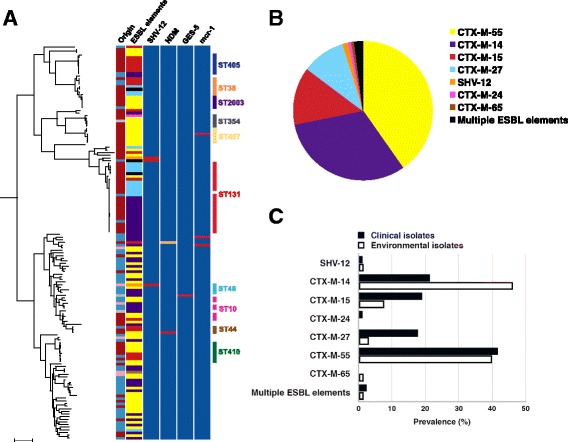
Distribution of ESBL, NDM and *mcr*-*1* genes in *E. coli* from East Thailand. **a** *Left*: maximum likelihood tree of 149 study isolate genomes based on SNPs in the core genome. Scale bar indicates ~ 10,000 SNPs. The columns describe the origin (*red*, clinical; *blue*, canal; *pink*, farm wastewater; *grey*, untreated hospital sewage), ESBL elements (*white*, absent; *yellow*, CTX-M-55; *purple*, CTX-M-14; *red*, CTX-M-15; *light blue*, CTX-M-27; *pink*, CTX-M-24; *brown*, CTX-M-65; *orange*, SHV-12; *black*, multiple ESBL elements) and data for SHV-12 (*blue*, absent; *red*, present), NDM (*blue*, absent; *red*, NDM-1; *orange*, NDM-5), GES-5 (*blue*, absent; *red*, present), and *mcr*-*1* (*blue*, absent; *red*, present). *Right*: the top ten multilocus sequence types. **b** Prevalence of ESBL elements (CTX-M and SHV-12 variants) in 149 study isolates. Three isolates contained multiple elements (CTX-M-15 and CTX-M-27, CTX-M-27 and CTX-M-55, and CTX-M-14 and SHV-12, respectively). **c** Percentage of each CTX-M variant and SHV-12 in 84 clinical versus 65 environmental isolates

Phenotypic antibiotic susceptibility and the presence of genes encoding antibiotic resistance were determined for all 149 ESBL-positive *E. coli*, the results of which are summarized in Figs. 2 and 3. All isolates were multidrug resistant (phenotypic resistance to three or more drug classes) [39]. Resistance to the carbapenem drugs was identified in two clinical isolates (resistant to ertapenem and meropenem), and one from hospital wastewater (intermediate resistance to ertapenem (MIC 0.75 μg/mL) but susceptible to meropenem (MIC 0.5 μg/mL)). The two carbapenem-resistant clinical isolates contained either New Delhi metallo-β-lactamase-1 (NDM-1; ST44) or NDM-5 (ST46), and the hospital wastewater isolate contained Guiana extended-spectrum β-lactamase-5 (GES-5; ST1585) (Fig. 3a).

The genetic basis for ESBL production is shown in Fig. 3. A total of 149 CTX-M elements identified in 147 isolates were resolved into six different elements, with CTX-M-55 (40.9%) the most common (Fig. 3b). SHV-12 type β-lactamase was present in three isolates (one clinical and two environmental), one of which was also positive for CTX-M-14. CTX-M-55 was the most prevalent CTX-M in clinical isolates (41.7%), whilst CTX-M-14 was the most prevalent in environmental isolates (44.6%). The proportions of CTX-M elements in clinical and environmental isolates are shown in Fig. 3c and a phylogeny of the CTX-M genes is shown in Additional file 3: Figure S2. Two variants of CTX-M-14 were identified that differed by one SNP and corresponded to CTX-M-14-1 (*n*  31; GenBank accession number JF701188) and CTX-M-14-48 (*n*  17; GenBank accession number AJ416341), respectively. In addition, two clinical isolates and one environmental isolate contained two different elements (Additional file 1: Table S1). Study isolates were screened for the presence of *mcr*-*1* and *mcr*-*2* encoding resistance to colistin, which detected *mcr*-*1* in three isolates (one clinical and two environmental from independent canals; Fig. 3a). Phenotypic resistance to colistin was confirmed using the E-test; MIC values of the three isolates were 4, 3 and 8 μg/mL.

### Phylogenetic analysis of ESBL-*E. coli* from Thailand

A maximum likelihood tree of the 149 ESBL-positive *E. coli* was created based on 227,154 SNPs in the 2682 core genes (Fig. 3a). The majority of clinical and environmental isolates resided on distinct branches of the phylogenetic tree, with environmental isolates showing a greater level of diversity than the clinical isolates. However, 11/72 STs contained both clinical and environmental isolates, and a pairwise comparison of SNPs in the core genes revealed that these were closely related (3–23 SNPs, median 9 SNPs) in 6/11 STs (ST131, ST2003, ST354, ST38, ST405 and ST410). Three pairs of clinical–environmental isolates differed by no more than five SNPs, whilst an additional 18 pairs of isolates differed by less than 25 SNPs. The environmental isolates were all from canals within 0.5 to 5.2 km of the hospital (median 2.3 km) with the exception of one isolate, which was taken from the hospital wastewater treatment system and was nine SNPs different from a clinical isolate.

The phylogeny of the 149 study genomes was contextualized by combining these with 514 *E. coli* genomes from a global collection. A maximum likelihood tree was created based on 222,900 SNPs in the 1983 core genes identified across the 663 isolates (Additional file 3: Figure S3). Thai *E. coli* isolates were distributed across the *E. coli* species population, suggesting a high level of diversity in the Thai *E. coli* population. Of note, one isolate from Thailand was two SNPs different from isolates from neighbouring Cambodia.

### Phylogenetic analysis of ST131 ESBL-producing *E. coli* in Thailand

**Fig. 4:**
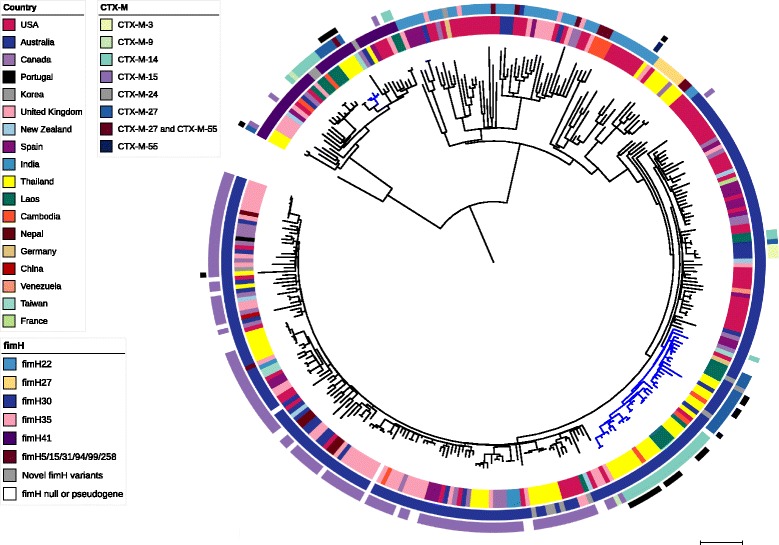
*E. coli* ST131 comparison. Maximum likelihood tree of the 26 study ST131 isolates and 312 global ST131 isolates mapped against the reference strain *E. coli* NTCC13441. Coloured rings from the inside out indicate country of origin, fimH type, CTX-M type and study isolates (*black*), respectively. Branches coloured *blue* indicate the major clusters of Thai isolates from this study (C2; 11 o’clock in the figure) or Thai isolates from this study clustered with phylogenetically related isolates from other countries (C1; 5 o’clock in the figure). *Scale bar* indicates 100 SNPs

Assignment of the 26 ST131 study isolates to fimH types revealed that the majority of Thai isolates belonged to H30 (76.9%) or H41 (19.2%). All 20 H30 isolates were resistant to ciprofloxacin and contained a CTX-M element (70% CTX-M-14, 25% CTX-M-27, 5% CTX-M-15). The majority of H30 isolates (19/20) belonged to the H30R subclone described by Price et al., with only one H30 isolate belonging to the H30Rx subclone. The ST131 study isolates were compared with a global collection of *E. coli* ST131 [18, 19, 29] (Fig. 4). The majority of Thai ST131 *E. coli* from this study belonged to two clusters (cluster 1 (*n* = 19) and cluster 2 (*n* = 4)). Cluster 1 belonged to H30R and contained isolates from Laos (*n* = 9), Cambodia (*n* = 3), Australia (*n* = 2), the USA (*n* = 2), Canada (*n* = 1), Germany (*n* = 1) and Taiwan (*n* = 1), interspersed with the 19 study isolates and two isolates from a previous study [29] from Thailand. An analysis of pairwise SNP differences revealed 14–92 SNPs between the Thai and global isolates in this cluster. Additionally, cluster 1 contained two environmental isolates from Thailand, obtained from independent canals, which were 19 and 38 SNPs from the nearest clinical isolates, both from Thailand. This cluster could be further divided into two groups based on the phylogeny and presence of CTX-M elements. One group (C1a) contained seven Thai isolates (five from this study), three Laotian, one Cambodian and two Australian isolates harbouring CTX-M-27, and an additional isolate from Laos harbouring CTX-M-24. The second group (C1b) contained 14 Thai, two Cambodian and five Laotian isolates harbouring CTX-M-14 (16 with CTX-M-14-1, three with CTX-M-14-48 and two with a novel CTX-M-14 variant). This suggests that these lineages may have acquired their CTX-M elements prior to spreading between countries. Additionally, both groups (C1a and C1b) contained CTX-M-negative isolates from Germany and/or the US that were basal to the CTX-M-positive isolates in the group. The remaining isolates in cluster 1 that did not belong to one of these two groups were a CTX-M-negative isolate from Taiwan and a single isolate from Canada that was CTX-M-14-positive and was located basal to the whole cluster. Cluster 2 consisted of four isolates from Thailand and belonged to the H41 sub-clone, which contained isolates from Laos (*n* = 8), Thailand (*n* = 7), the United Kingdom (*n* = 7), the USA (*n* = 5), Cambodia (*n* = 2), Australia (*n* = 2), Canada (*n* = 2), New Zealand (*n* = 1), Spain (*n* = 1) and Taiwan (*n* = 1) (Fig. 4). The four Thai isolates residing in cluster 2 contained CTX-M-27 alone (three isolates), or both CTX-M-27 and CTX-M-55 (one isolate).

### Characterisation of *mcr*-*1* and carbapenamase-encoding plasmids

We investigated the genetic context of the colistin and carbapenemase resistance genes detected in the study isolates by comparing the contigs containing these genes to the GenBank database. The highest matches to the contigs containing *mcr*-*1* were equal matches to *E. coli* plasmids pECJS-59-244, pS38 and EC2-4 for one of the canal isolates; and *S. enterica* subsp. diarizonae strain 11-01854 and 11-01853 plasmids for the clinical isolate and the second canal isolate (Additional file 4: Table S3). The three highly related *E. coli* plasmids have been reported previously as carrying the *mcr*-*1* gene, and were isolated in China, Switzerland and Malaysia. Additionally, it has been reported that *mcr*-*1* in three *E. coli* from commercial farms in China were carried on plasmids with a similar backbone to plasmid 11-01854 [40]. The contig containing NDM-1 was best matched to *E. coli* plasmid pNDM-ECS01, which has been described previously as carrying NDM-1 in ST131 *E. coli* and *Klebsiella pneumoniae* in Thailand [41]. The contig containing NDM-5 had equal matches to *E. coli* plasmids pC06114_1 and pGUE-NDM, which carry NDM-1, and *K. pneumoniae* plasmids pCC1409-1 and pCC1410-1, which carry NDM-5 and were identified in Korea after transfer of a patient from the United Arab Emirates [42]. Finally, the contig containing GES-5 was best matched to *E. coli* plasmid pHKU1, which was described in the context of fosfomycin resistance in *E. coli* and to our knowledge has not been reported previously to carry GES-5. However, only 40% of the contig matched plasmid pHKU1, indicating differences in the genetic content (Additional file 4: Table S3). Overall these results indicate that plasmids carrying *mcr*-*1* in Thailand may be globally disseminated, and those carrying carbapenamase resistance genes could have originated from other countries in Asia or the Middle East. The finding of *mcr*-*1* on a potentially novel plasmid indicates further dissemination of *mcr*-*1* in the plasmid population.

### Virulence genes

Of the 284 virulence genes investigated, 153 were detected at least once in the 149 study isolates. Analysis of the distribution of virulence factors across STs indicated that there was a higher prevalence of virulence genes in ST131. To further examine this, we compared the prevalence of each virulence factor in ST131 (*n* = 26) and non-ST131 (*n* = 123). A total of 36 virulence genes were significantly more common in ST131 (all < 0.0001 except for *fim* genes, *p* < 0.05) (Additional file 3: Figure S4); 35 of these associations have not been reported previously (the exception being *sat*) [43–46]. Twelve virulence genes were significantly less common in ST131 isolates (*p* value < 0.05), including four genes (*entD*, *espX1*, *espX5* and *espL1*) present in over 79% of non-ST131 isolates but absent in ST131 isolates (Additional file 3: Figure S4).

## Discussion

This study has demonstrated that Thai ESBL-positive *E. coli* isolates are genetically diverse, with isolates dispersed throughout a phylogenetic tree containing a global collection of *E. coli*. Thai isolates cultured from the environment were more genetically diverse than those from Thai patients, and a core genome comparison indicated that ESBL-positive *E. coli* from humans and the environment (including farms) were broadly distinct, although there was evidence for some highly related bacterial pairs. All of the Thai ESBL-positive *E. coli* isolates were multidrug resistant, including high rates of resistance to aminoglycosides, fluoroquinolones and trimethoprim. A small minority of isolates were also resistant to the carbapenem drugs (encoded by NDM-1, NDM-5 or GES-5) or to colistin (encoded by *mcr*-*1*), but no isolates were resistant to both the carbapenem drugs and colistin. Of note, isolates resistant to the carbapenem drugs were only isolated from clinical samples or a hospital sewer, while isolates resistant to colistin were identified from both clinical samples and canal water.

The isolation of NDM-1-positive *E. coli* from Thailand has been reported previously from urine samples cultured from patients with urinary tract infection at Srinagarind Hospital, Khon Kaen province in 2011 [47]. To our knowledge, this is the first report of both NDM-5 and GES-5 in Thailand, but NDM-5-positive *E. coli* has been isolated from humans in numerous countries, including the United Kingdom in 2011 [48], and in China, Algeria, Japan, USA, Denmark, Australia, Spain, Egypt and South Korea between 2014 and 2016 [49–56]. NDM-5-positive *E. coli* has also been identified from a domestic animal in Algeria [57]. GES-5-positive *E. coli* was first documented in Greece in 2004 in a clinical isolate with low level resistance to carbapenem drugs [58]. The first report of *mcr*-*1*-positive *E. coli* was from China in 2015 but there is increasing evidence that the *mcr*-*1* gene had been circulating in Asia prior to this, including in Thailand based on the retrospective identification of *mcr*-*1*-positive *E. coli* that was originally isolated in 2011 [4, 37]. Blast comparisons performed during our study of contigs containing *mcr*-*1* and genes encoding carbapenem resistance were consistent with this being plasmid-mediated. Genetic characterisation of CTX-M genes was notable for the diversity in elements identified, with six elements identified but with CTX-M-55 being the most common and present in clinical and environmental isolates. By contrast, the proportion of other elements (for example, CTX-M-15 and CTX-M-27) varied considerably between human and environmental isolates.

*E. coli* ST131 was the predominant clone associated with human disease in Thailand and accounted for almost one-third of isolates, although was uncommonly isolated from the environment. This lineage is frequently multidrug-resistant, has become globally disseminated and is a well-known cause of human disease [59, 60]. We identified 36 virulence factors that were over-represented in ST131 compared with non-ST131 isolates. This comparison should be interpreted with caution since this was underpowered (26 ST131 isolates versus 123 non-ST131 isolates), and the comparison of a single clone with a genetically heterogeneous second group is potentially problematic. However, differences in gene contents between ST131 and other lineages is consistent with previous studies, which have reported over-representation in ST131 of genes associated with adhesion (*afa/dra*, *fimH*, *papA* and *iha*,), invasin (*ibeA*), iron acquisition (*fyuA* and *iutA*), toxins (*sat* and *aer*), protectins (*kpsMII*, *kpsMIII*, *K2*, *K5* and *ompT*) and other functions (*usp*, *traT* and *malX*) [43–46, 61].

Our findings add to the increasing body of evidence that ESBL-positive *E. coli* is ubiquitous in Thailand. A recent study reported that ESBL-producing *E. coli* could be isolated from three-quarters of healthy Thai people tested and was also common in livestock, which suggests that this has become established in humans and their food chain [7]. There is also direct evidence for human consumption of food containing ESBL-producing *E. coli*, which was isolated from pre-cooked and cooked meat collected from markets in Bangkok, Thailand in 2012–2013 [7]. Furthermore, there is evidence for global dissemination since nearly 20% of a sample consisting of a range of fresh vegetables imported into Switzerland from Thailand in 2014 was positive for ESBL-producing *E. coli* [62]. This is having an impact on human health in Thailand based on the findings of a retrospective study conducted in nine public hospitals in northeast Thailand [63]. The proportions of community-acquired *E. coli* bacteraemia caused by *E. coli* non-susceptible to extended-spectrum cephalosporins rose from 5 to 23% between 2004 and 2010, while the proportions of healthcare-associated and hospital-acquired *E. coli* bacteraemia caused by *E. coli* non-susceptible to extended-spectrum cephalosporins were high (44 and 52%, respectively) with no significant change over time [63]. Mortality was higher in patients with multidrug-resistant *E. coli* bacteraemia compared with non-multidrug-resistant *E. coli* bacteraemia [63], indicating the human cost of this problem.

## Conclusions

Our genome-based study adds to the epidemiological and phenotypic data for ESBL-positive *E. coli* in Thailand. There was some evidence for highly related *E. coli* between infected patients and their immediate environment, and we identified a plethora of genes encoding drug resistance to broad-spectrum antibiotics used to treat Gram-negative infections. The extent to which ESBL-positive *E. coli* is distributed in humans, the environment and the food chain, the diversity of elements encoding ESBL and the epidemiological rise in the proportion of multidrug-resistant *E. coli* causing human disease indicates that this has gathered pace over the past decade. ESBL genes likely provide a fitness advantage to *E. coli* belonging to numerous different lineages, both in the environment and in clinical settings. Furthermore, these genetic elements are likely to be present in a range of additional Gram-negative bacterial species. Reversing the clock on ESBL in this setting may prove extremely difficult, but our finding of a comparatively low prevalence of mobile genes encoding resistance to the carbapenem drugs and colistin suggests that efforts are now required to prevent these from also becoming ubiquitously distributed.

## Additional files

Additional file 1: Table S1. Isolate details. (XLSX 15 kb)

Additional file 2: Table S2. Sequence quality metrics. (XLSX 495 kb)

Additional file 3: Figure S1. Predominant STs of ESBL-producing *E. coli* isolated from clinical and environmental origins in Thailand. Graph showing the prevalence of the eight most frequently identified STs among each of the clinical or environmental Thai ESBL-producing *E. coli* isolates, split by source of isolation. The total adds up to 12 since four of the top eight most frequent in each category did not overlap. The presence of all isolates in each of the 12 STs is shown. **Figure S2.** Phylogeny of CTX-M genes identified in Thai ESBL-producing *E. coli*. Phylogeny of the seven CTX-M gene variants identified in the 149 study genomes. **Figure S3.** Phylogeny of Thai ESBL-producing *E. coli* in a global context. Maximum-likelihood tree of the 149 study genomes and 514 global genomes based on 222,900 SNPs in the 1983 core genes. *Inner ring* shows origin of isolate, *middle ring* shows country of isolation and *outer ring* indicates the study isolates (*black*). *Scale bar* indicates ~ 20,000 SNPs. **Figure S4.** Virulence gene comparison between ST131 and non-ST131 isolates. Graph showing the prevalence of virulence factors in ST131 (*n*  26) compared to non-ST131 (*n*  123) in the study isolates. Using Fisher’s exact test at a *p* value of 0.05, only those factors over-represented between the two groups are shown. (DOCX 2381 kb)

Additional file 4: Table S3. Genetic context of *mcr*-*1* and carbapenem resistance genes. (XLSX 9 kb)

## Abbreviations

CLSI: Clinical and Laboratory Standards Institute
CTX-M: Cefotaxime-Munich
ENA: European Nucleotide Archive
ESBL: Extended-spectrum β-lactamase
E-test: Epsilometer test
EUCAST: European Committee on Antimicrobial Susceptibility Testing
GES: Guiana extended-spectrum β-lactamase
MALDI-TOF MS: Matrix assisted laser desorption ionization-time of flight mass spectrometry
*mcr*-*1*: mobile colistin resistance gene-1
MIC: Minimum inhibitory concentration
MLST: Multilocus sequence typing
NDM: New Delhi metallo-β-lactamase
SHV: Sulfhydryl variable
SNP: Single nucleotide polymorphism
ST: Sequence type

## References

[1] Pitout JD, Laupland KB (2008). Extended-spectrum beta-lactamase-producing *Enterobacteriaceae*: an emerging public-health concern. Lancet Infect Dis.

[2] Schwaber MJ, Navon-Venezia S, Kaye KS, Ben-Ami R, Schwartz D, Carmeli Y (2006). Clinical and economic impact of bacteremia with extended-spectrum beta-lactamase-producing *Enterobacteriaceae*. Antimicrob Agents Chemother.

[3] Nordmann P, Naas T, Poirel L (2011). Global spread of carbapenemase-producing *Enterobacteriaceae*. Emerg Infect Dis.

[4] Schwarz S, Johnson AP (2016). Transferable resistance to colistin: a new but old threat. J Antimicrob Chemother.

[5] Xavier BB, Lammens C, Ruhal R, Kumar-Singh S, Butaye P, Goossens H, Malhotra-Kumar S. Identification of a novel plasmid-mediated colistin-resistance gene, *mcr-2*, in Escherichia coli, Belgium, June 2016. Euro Surveill*.* 2016;21(27):30280. 10.2807/1560-7917.ES.2016.21.27.30280 27416987

[6] Oh S, Buddenborg S, Yoder-Himes DR, Tiedje JM, Konstantinidis KT (2012). Genomic diversity of *Escherichia* isolates from diverse habitats. PLoS One.

[7] Boonyasiri A, Tangkoskul T, Seenama C, Saiyarin J, Tiengrim S, Thamlikitkul V (2014). Prevalence of antibiotic resistant bacteria in healthy adults, foods, food animals, and the environment in selected areas in Thailand. Pathog Glob Health.

[8] Ma J, Liu JH, Lv L, Zong Z, Sun Y, Zheng H, Chen Z, Zeng ZL (2012). Characterization of extended-spectrum beta-lactamase genes found among *Escherichia coli* isolates from duck and environmental samples obtained on a duck farm. Appl Environ Microbiol.

[9] Tian GB, Wang HN, Zhang AY, Zhang Y, Fan WQ, Xu CW, Zeng B, Guan ZB, Zou LK (2012). Detection of clinically important beta-lactamases in commensal *Escherichia coli* of human and swine origin in western China. J Med Microbiol.

[10] Tamang MD, Nam HM, Gurung M, Jang GC, Kim SR, Jung SC, Park YH, Lim SK (2013). Molecular characterization of CTX-M beta-lactamase and associated addiction systems in *Escherichia coli* circulating among cattle, farm workers, and the farm environment. Appl Environ Microbiol.

[11] Blaak H, van Hoek AH, Hamidjaja RA, van der Plaats RQ, Kerkhof-de Heer L, de Roda Husman AM, Schets FM. Distribution, numbers, and diversity of ESBL-producing E. coli in the poultry farm environment. PLoS One. 2015;10(8):e0135402. 10.1371/journal.pone.0135402 PMC4536194 26270644

[12] Diab M, Hamze M, Madec JY, Haenni M (2016). High prevalence of non-ST131 CTX-M-15-producing *Escherichia coli* in healthy cattle in Lebanon. Microb Drug Resist.

[13] Seiffert SN, Hilty M, Perreten V, Endimiani A (2013). Extended-spectrum cephalosporin-resistant Gram-negative organisms in livestock: an emerging problem for human health?. Drug Resist Updat.

[14] Hartmann A, Locatelli A, Amoureux L, Depret G, Jolivet C, Gueneau E, Neuwirth C (2012). Occurrence of CTX-M producing *Escherichia coli* in soils, cattle, and farm environment in France (Burgundy Region). Front Microbiol..

[15] Xi C, Zhang Y, Marrs CF, Ye W, Simon C, Foxman B, Nriagu J (2009). Prevalence of antibiotic resistance in drinking water treatment and distribution systems. Appl Environ Microbiol.

[16] Koser CU, Ellington MJ, Peacock SJ (2014). Whole-genome sequencing to control antimicrobial resistance. Trends Genet.

[17] Luo C, Walk ST, Gordon DM, Feldgarden M, Tiedje JM, Konstantinidis KT (2011). Genome sequencing of environmental *Escherichia coli* expands understanding of the ecology and speciation of the model bacterial species. Proc Natl Acad Sci U S A.

[18] Price LB, Johnson JR, Aziz M, Clabots C, Johnston B, Tchesnokova V, Nordstrom L, Billig M, Chattopadhyay S, Stegger M, et al. The epidemic of extended-spectrum beta-lactamase-producing *Escherichia coli* ST131 is driven by a single highly pathogenic subclone, H30-Rx. mBio. 2013;4(6):e00377–13. 10.1128/mBio.00377-13 PMC3870262 24345742

[19] Petty NK, Ben Zakour NL, Stanton-Cook M, Skippington E, Totsika M, Forde BM, Phan MD, Gomes Moriel D, Peters KM, Davies M (2014). Global dissemination of a multidrug resistant *Escherichia coli* clone. Proc Natl Acad Sci U S A.

[20] Matsumura Y, Pitout JD, Gomi R, Matsuda T, Noguchi T, Yamamoto M, Peirano G, DeVinney R, Bradford PA, Motyl MR (2016). Global *Escherichia coli* sequence type 131 clade with *blaCTX-M-27* gene. Emerg Infect Dis.

[21] Trongjit S, Angkittitrakul S, Chuanchuen R (2016). Occurrence and molecular characteristics of antimicrobial resistance of *Escherichia coli* from broilers, pigs and meat products in Thailand and Cambodia provinces. Microbiol Immunol.

[22] Runcharoen C, Moradigaravand D, Blane B, Paksanont S, Thammachote J, Anun S, Parkhill J, Chantratita N, Peacock SJ (2017). Whole genome sequencing reveals high-resolution epidemiological links between clinical and environmental *Klebsiella pneumoniae*. Genome Med.

[23] Raven KE, Reuter S, Gouliouris T, Reynolds R, Russell JE, Brown NM, Torok ME, Parkhill J, Peacock SJ (2016). Genome-based characterization of hospital-adapted *Enterococcus faecalis* lineages. Nat Microbiol.

[24] Page AJ, De Silva N, Hunt M, Quail MA, Parkhill J, Harris SR, Otto TD, Keane JA (2016). Robust high-throughput prokaryote *de novo* assembly and improvement pipeline for Illumina data. Microb Genom.

[25] Page AJ, Cummins CA, Hunt M, Wong VK, Reuter S, Holden MT, Fookes M, Falush D, Keane JA, Parkhill J (2015). Roary: rapid large-scale prokaryote pan-genome analysis. Bioinformatics.

[26] de Been M, Lanza VF, de Toro M, Scharringa J, Dohmen W, Du Y, Hu J, Lei Y, Li N, Tooming-Klunderud A (2014). Dissemination of cephalosporin resistance genes between *Escherichia coli* strains from farm animals and humans by specific plasmid lineages. PLoS Genet.

[27] von Mentzer A, Connor TR, Wieler LH, Semmler T, Iguchi A, Thomson NR, Rasko DA, Joffre E, Corander J, Pickard D (2014). Identification of enterotoxigenic *Escherichia coli* (ETEC) clades with long-term global distribution. Nat Genet.

[28] Stoesser N, Sheppard AE, Moore CE, Golubchik T, Parry CM, Nget P, Saroeun M, Day NP, Giess A, Johnson JR (2015). Extensive within-host diversity in fecally carried extended-spectrum beta-lactamase producing *Escherichia coli* isolates: implications for transmission Analyses. J Clin Microbiol.

[29] Stoesser N, Sheppard AE, Pankhurst L, De Maio N, Moore CE, Sebra R, Turner P, Anson LW, Kasarskis A, Batty EM, et al. Evolutionary history of the global emergence of the *Escherichia coli* epidemic clone ST131. mBio. 2016;7(2):e02162. 10.1128/mBio.02162-15 PMC4807372 27006459

[30] Brodrick HJ, Kathy RE, Kallonen T, Jamrozy D, Blane B, Brown NM, Martin V, Torok ME, Parkhill J, Peacock SJ. Longitudinal genomic surveillance of multidrug-resistant *Escherichia coli* carriage in a longterm care facility in the United Kingdom. Genome Med. 2017;9(1):70. 10.1186/s13073-017-0457-6 PMC5525225 28738847

[31] Croucher NJ, Page AJ, Connor TR, Delaney AJ, Keane JA, Bentley SD, Parkhill J, Harris SR (2015). Rapid phylogenetic analysis of large samples of recombinant bacterial whole genome sequences using Gubbins. Nucleic Acids Res.

[32] Price LB, Johnson JR, Aziz M, Clabots C, Johnston B, Tchesnokova V, Nordstrom L, Billig M, Chattopadhyay S, Stegger M et al. The epidemic of extended-spectrum beta-lactamase-producing Escherichia coli ST131 is driven by a single highly pathogenic subclone, H30-Rx. mBio. 2013;4(6):e00377–13. 10.1128/mBio.00377-13 PMC3870262 24345742

[33] Stoesser N, Sheppard AE, Pankhurst L, De Maio N, Moore CE, Sebra R, Turner P, Anson LW, Kasarskis A, Batty EM et al. Evolutionary history of the global emergence of the Escherichia coli epidemic clone ST131. mBio. 2016;7(2):e02162. 10.1128/mBio.02162-15 PMC4807372 27006459

[34] Weissman SJ, Johnson JR, Tchesnokova V, Billig M, Dykhuizen D, Riddell K, Rogers P, Qin X, Butler-Wu S, Cookson BT (2012). High-resolution two-locus clonal typing of extraintestinal pathogenic *Escherichia coli*. Appl Environ Microbiol.

[35] Paul S, Linardopoulou EV, Billig M, Tchesnokova V, Price LB, Johnson JR, Chattopadhyay S, Sokurenko EV (2013). Role of homologous recombination in adaptive diversification of extraintestinal *Escherichia coli*. J Bacteriol.

[36] Zankari E, Hasman H, Cosentino S, Vestergaard M, Rasmussen S, Lund O, Aarestrup FM, Larsen MV (2012). Identification of acquired antimicrobial resistance genes. J Antimicrob Chemother.

[37] Liu YY, Wang Y, Walsh TR, Yi LX, Zhang R, Spencer J, Doi Y, Tian G, Dong B, Huang X (2016). Emergence of plasmid-mediated colistin resistance mechanism MCR-1 in animals and human beings in China: a microbiological and molecular biological study. Lancet Infect Dis.

[38] Price MN, Dehal PS, Arkin AP (2010). FastTree 2--approximately maximum-likelihood trees for large alignments. PLoS One.

[39] Magiorakos AP, Srinivasan A, Carey RB, Carmeli Y, Falagas ME, Giske CG, Harbarth S, Hindler JF, Kahlmeter G, Olsson-Liljequist B (2012). Multidrug-resistant, extensively drug-resistant and pandrug-resistant bacteria: an international expert proposal for interim standard definitions for acquired resistance. Clin Microbiol Infect.

[40] Wang Y, Zhang R, Li J, Wu Z, Yin W, Schwarz S, Tyrrell JM, Zheng Y, Wang S, Shen Z (2017). Comprehensive resistome analysis reveals the prevalence of NDM and MCR-1 in Chinese poultry production. Nat Microbiol..

[41] Netikul T, Sidjabat HE, Paterson DL, Kamolvit W, Tantisiriwat W, Steen JA, Kiratisin P (2014). Characterization of an IncN2-type blaNDM-(1)-carrying plasmid in *Escherichia coli* ST131 and *Klebsiella pneumoniae* ST11 and ST15 isolates in Thailand. J Antimicrob Chemother.

[42] Cho SY, Huh HJ, Baek JY, Chung NY, Ryu JG, Ki CS, Chung DR, Lee NY, Song JH (2015). *Klebsiella pneumoniae* co-producing NDM-5 and OXA-181 carbapenemases. South Korea. Emerg Infect Dis..

[43] Johnson JR, Porter SB, Zhanel G, Kuskowski MA, Denamur E (2012). Virulence of *Escherichia coli* clinical isolates in a murine sepsis model in relation to sequence type ST131 status, fluoroquinolone resistance, and virulence genotype. Infect Immun.

[44] Johnson JR, Urban C, Weissman SJ, Jorgensen JH (2012). Lewis 2nd JS, Hansen G, Edelstein PH, Robicsek A, Cleary T, Adachi J, et al. Molecular epidemiological analysis of *Escherichia coli* sequence type ST131 (O25:H4) and *blaCTX-M-15* among extended-spectrum beta-lactamase producing *E. coli* from the United States, 2000 to 2009. Antimicrob Agents Chemother.

[45] Colpan A, Johnston B, Porter S, Clabots C, Anway R, Thao L, Kuskowski MA, Tchesnokova V, Sokurenko EV, Johnson JR (2013). *Escherichia coli* sequence type 131 (ST131) subclone H30 as an emergent multidrug-resistant pathogen among US veterans. Clin Infect Dis.

[46] Olesen B, Hansen DS, Nilsson F, Frimodt-Moller J, Leihof RF, Struve C, Scheutz F, Johnston B, Krogfelt KA, Johnson JR (2013). Prevalence and characteristics of the epidemic multiresistant *Escherichia coli* ST131 clonal group among extended-spectrum beta-lactamase-producing *E. coli* isolates in Copenhagen, Denmark. J Clin Microbiol.

[47] Rimrang B, Chanawong A, Lulitanond A, Wilailuckana C, Charoensri N, Sribenjalux P, Phumsrikaew W, Wonglakorn L, Kerdsin A, Chetchotisakd P (2012). Emergence of NDM-1 and IMP-14a-producing *Enterobacteriaceae* in Thailand. J Antimicrob Chemother.

[48] Hornsey M, Phee L, Wareham DW (2011). A novel variant, NDM-5, of the New Delhi metallo-beta-lactamase in a multidrug-resistant *Escherichia coli* ST648 isolate recovered from a patient in the United Kingdom. Antimicrob Agents Chemother.

[49] Yang P, Xie Y, Feng P, Zong Z (2014). *blaNDM-5* carried by an IncX3 plasmid in *Escherichia coli* sequence type 167. Antimicrob Agents Chemother.

[50] Nakano R, Nakano A, Hikosaka K, Kawakami S, Matsunaga N, Asahara M, Ishigaki S, Furukawa T, Suzuki M, Shibayama K (2014). First report of metallo-beta-lactamase NDM-5-producing *Escherichia coli* in Japan. Antimicrob Agents Chemother.

[51] Sassi A, Loucif L, Gupta SK, Dekhil M, Chettibi H, Rolain JM (2014). NDM-5 carbapenemase-encoding gene in multidrug-resistant clinical isolates of *Escherichia coli* from Algeria. Antimicrob Agents Chemother.

[52] Epstein L, Hunter JC, Arwady MA, Tsai V, Stein L, Gribogiannis M, Frias M, Guh AY, Laufer AS, Black S (2014). New Delhi metallo-beta-lactamase-producing carbapenem-resistant *Escherichia coli* associated with exposure to duodenoscopes. JAMA.

[53] Hammerum AM, Littauer P, Hansen F (2015). Detection of *Klebsiella pneumoniae* co-producing NDM-7 and OXA-181, *Escherichia coli* producing NDM-5 and *Acinetobacter baumannii* producing OXA-23 in a single patient. Int J Antimicrob Agents.

[54] Wailan AM, Paterson DL, Caffery M, Sowden D, Sidjabat HE (2015). Draft genome sequence of NDM-5-producing *Escherichia coli* sequence type 648 and genetic context of *blaNDM-5* in Australia. Genome Announc.

[55] Gamal D, Fernandez-Martinez M, El-Defrawy I, Ocampo-Sosa AA, Martinez-Martinez L (2016). First identification of NDM-5 associated with OXA-181 in *Escherichia coli* from Egypt. Emerg Microbes Infect..

[56] Park M, Park SD, Lee MH, Kim SH, Lim K, Lee G, Woo HJ, Kim HW, Yang JY, Eom YB (2016). The first report of NDM-5-producing uropathogenic *Escherichia coli* isolates in South Korea. Diagn Microbiol Infect Dis.

[57] Yousfi M, Mairi A, Bakour S, Touati A, Hassissen L, Hadjadj L, Rolain JM (2015). First report of NDM-5-producing *Escherichia coli* ST1284 isolated from dog in Bejaia. Algeria. New Microbes New Infect..

[58] Vourli S, Giakkoupi P, Miriagou V, Tzelepi E, Vatopoulos AC, Tzouvelekis LS (2004). Novel GES/IBC extended-spectrum beta-lactamase variants with carbapenemase activity in clinical enterobacteria. FEMS Microbiol Lett.

[59] Rogers BA, Sidjabat HE, Paterson DL (2011). *Escherichia coli* O25b-ST131: a pandemic, multiresistant, community-associated strain. J Antimicrob Chemother.

[60] Pitout JD, Campbell L, Church DL, Gregson DB, Laupland KB (2009). Molecular characteristics of travel-related extended-spectrum beta-lactamase-producing *Escherichia coli* isolates from the Calgary Health Region. Antimicrob Agents Chemother.

[61] Hussain A, Ranjan A, Nandanwar N, Babbar A, Jadhav S, Ahmed N (2014). Genotypic and phenotypic profiles of *Escherichia coli* isolates belonging to clinical sequence type 131 (ST131), clinical non-ST131, and fecal non-ST131 lineages from India. Antimicrob Agents Chemother.

[62] Zurfluh K, Nuesch-Inderbinen M, Morach M, Zihler Berner A, Hachler H, Stephan R (2015). Extended-spectrum-beta-lactamase-producing *Enterobacteriaceae* isolated from vegetables imported from the Dominican Republic, India, Thailand, and Vietnam. Appl Environ Microbiol.

[63] Lim C, Takahashi E, Hongsuwan M, Wuthiekanun V, Thamlikitkul V, Hinjoy S, Day NP, Peacock SJ, Limmathurotsakul D (2016). Epidemiology and burden of multidrug-resistant bacterial infection in a developing country. eLife..

