# Supplementary material for: Whole genome sequencing of ESBL-producing Escherichia coli isolated from patients, farm waste and canals in Thailand
Source: Genome Med. 2017 Sep 6;9:81. doi: 10.1186/s13073-017-0471-8 (PMC5588602; doi:10.1186/s13073-017-0471-8)
Supplement: Supplementary file 3 — Predominant STs of ESBL-producing E. coli isolated from clinical and environmental origins in Thailand. Graph showing the prevalence of the eight most frequently identified STs among each of the clinical or environmental Thai ESBL-producing E. coli isolates, split by source of isolation. The total adds up to 12 since four of the top eight most frequent in each category did not overlap. The presence of all isolates in each of the 12 STs is shown. Figure S2. Phylogeny of CTX-M genes identified in Thai ESBL-producing E. coli. Phylogeny of the seven CTX-M gene variants identified in the 149 study genomes. Figure S3. Phylogeny of Thai ESBL-producing E. coli in a global context. Maximum-likelihood tree of the 149 study genomes and 514 global genomes based on 222,900 SNPs in the 1983 core genes. Inner ring shows origin of isolate, middle ring shows country of isolation and outer ring indicates the study isolates (black). Scale bar indicates ~ 20,000 SNPs. Figure S4. Virulence gene comparison between ST131 and non-ST131 isolates. Graph showing the prevalence of virulence factors in ST131 (n 26) compared to non-ST131 (n 123) in the study isolates. Using Fisher’s exact test at a p value of 0.05, only those factors over-represented between the two groups are shown. (DOCX 2381 kb) [file 13073_2017_471_MOESM3_ESM.docx]

**SUPPLEMENTARY FIGURES**

**Whole genome sequencing of ESBL-producing *E. coli* isolated from patients, farm waste and canals in Thailand**

Chakkaphan Runcharoen, Kathy E. Raven, Sandra Reuter, Teemu Kallonen, Suporn Paksanont, Jeeranan Thammachote, Suthatip Anun, Beth Blane, Julian Parkhill, Sharon J. Peacock, Narisara Chantratita


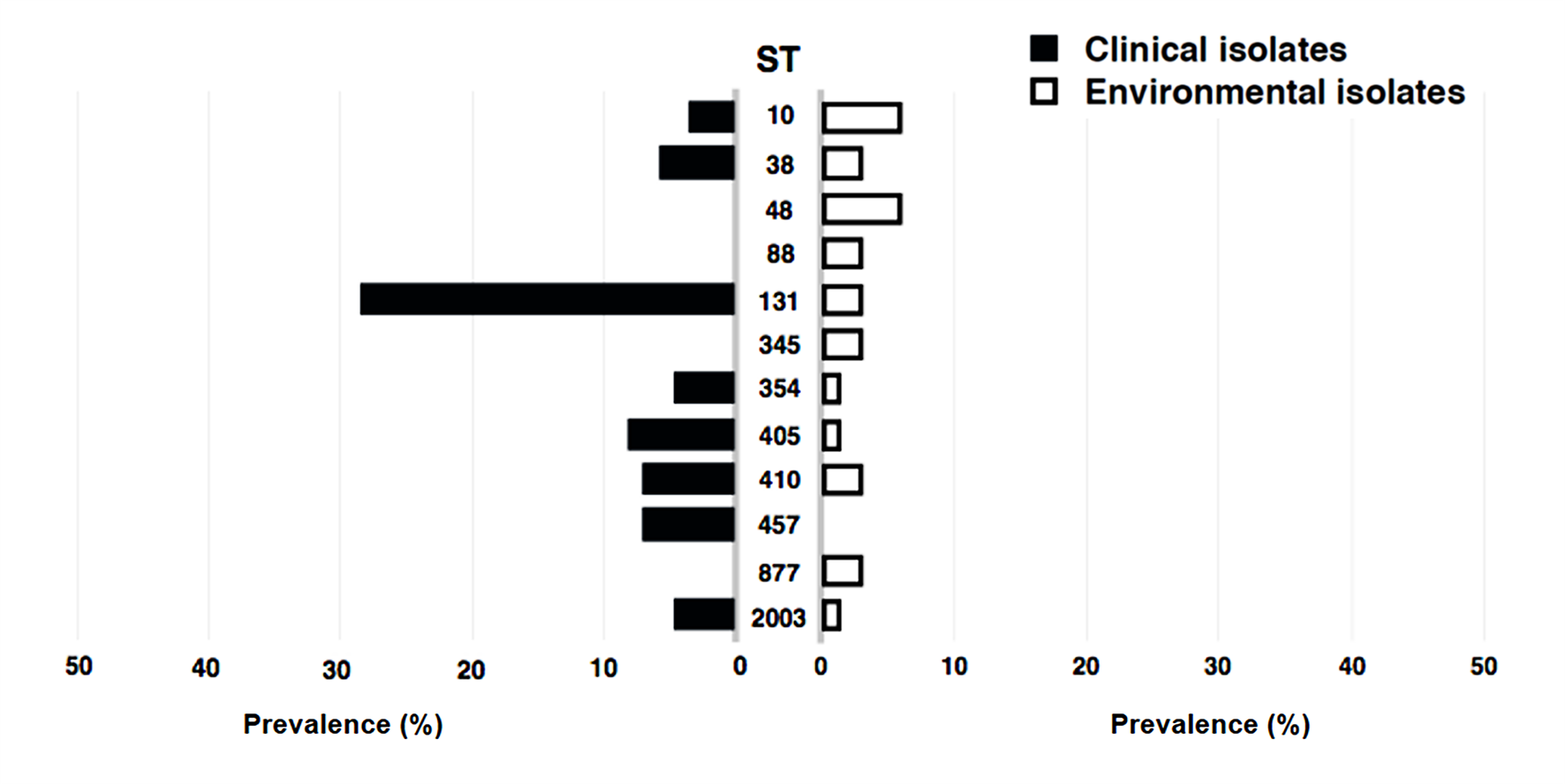


Figure S1 Predominant STs of ESBL-producing *E. coli* isolated from clinical and environmental origins in Thailand. Graph showing the prevalence of the eight most frequently identified STs among each of the clinical or environmental Thai ESBL-producing *E. coli* isolates, split by source of isolation. The total adds up to twelve since four of the top eight most frequent in each category did not overlap. The presence of all isolates in each of the 12 STs are shown.


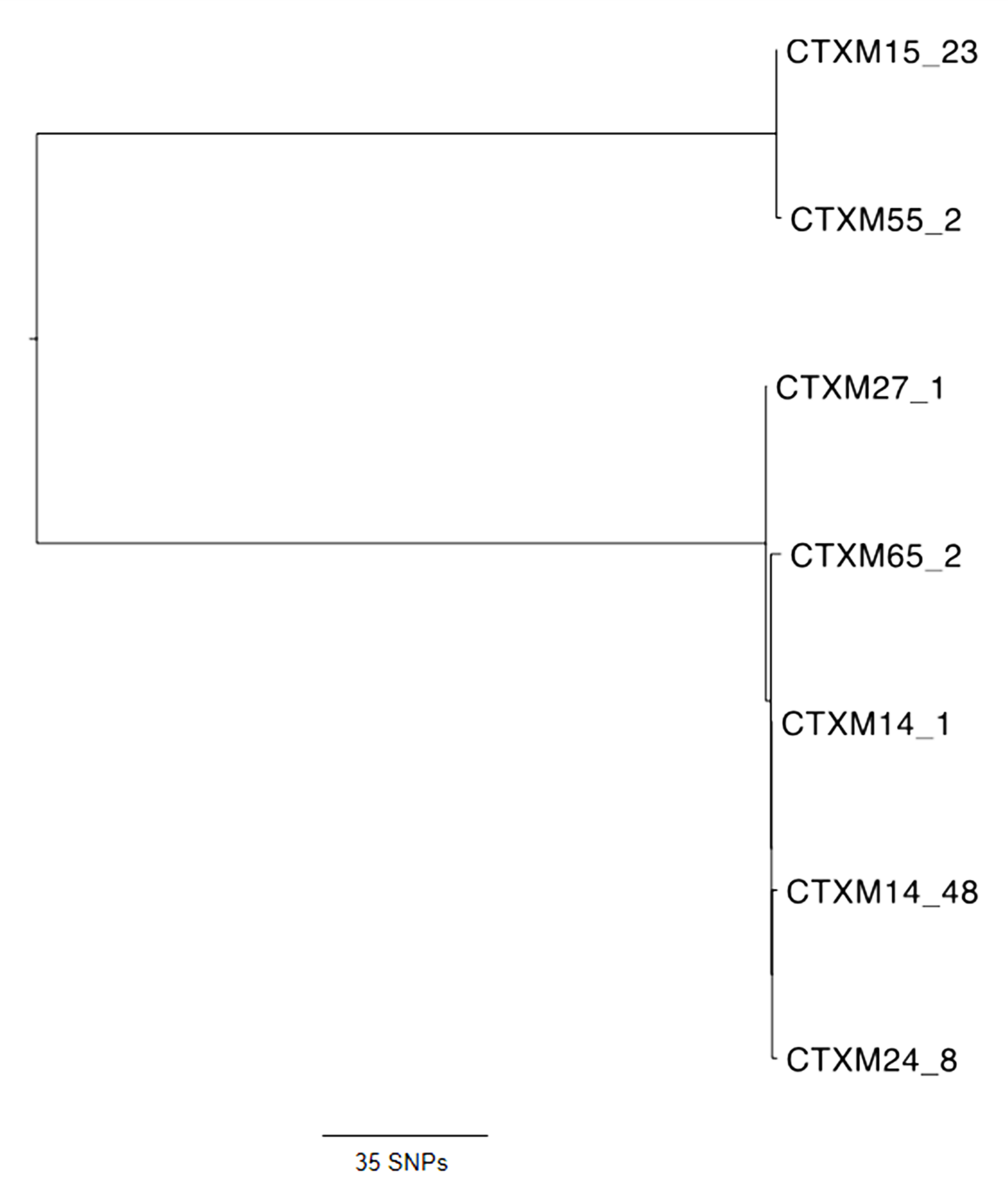


Figure S2 Phylogeny of CTX-M genes identified in Thai ESBL-producing *E. coli*. Phylogeny of the seven CTX-M gene variants identified in the 149 study genomes.


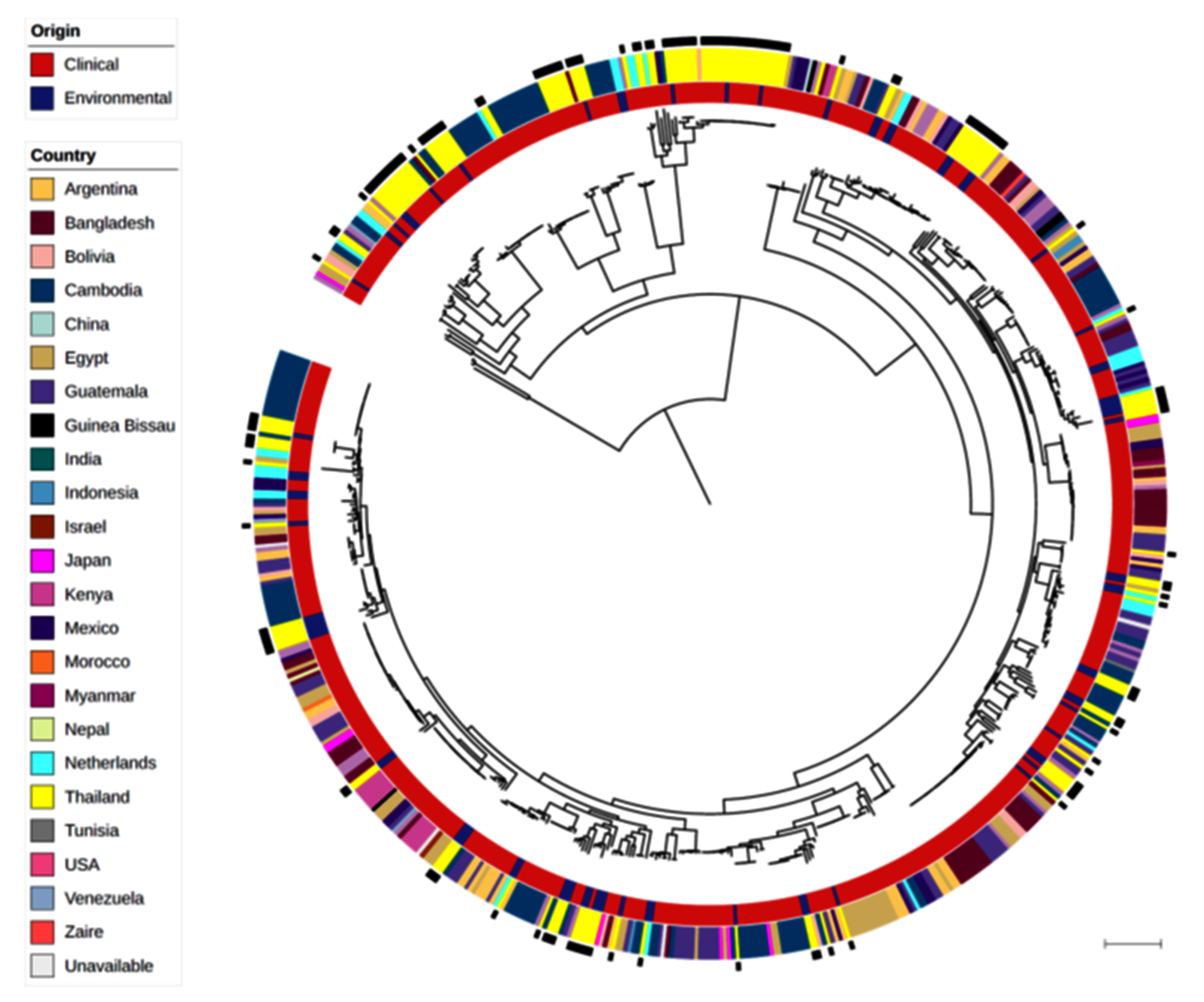


Figure S3 Phylogeny of Thai ESBL-producing *E. coli* in a global context. Maximum-likelihood tree of the 149 study genomes and 514 global genomes based on 222,900 SNPs in the 1,983 core genes. Inner ring shows origin of isolate, middle ring shows country of isolation and outer ring indicates the study isolates (black). Scale bar indicates ~20,000 SNPs.


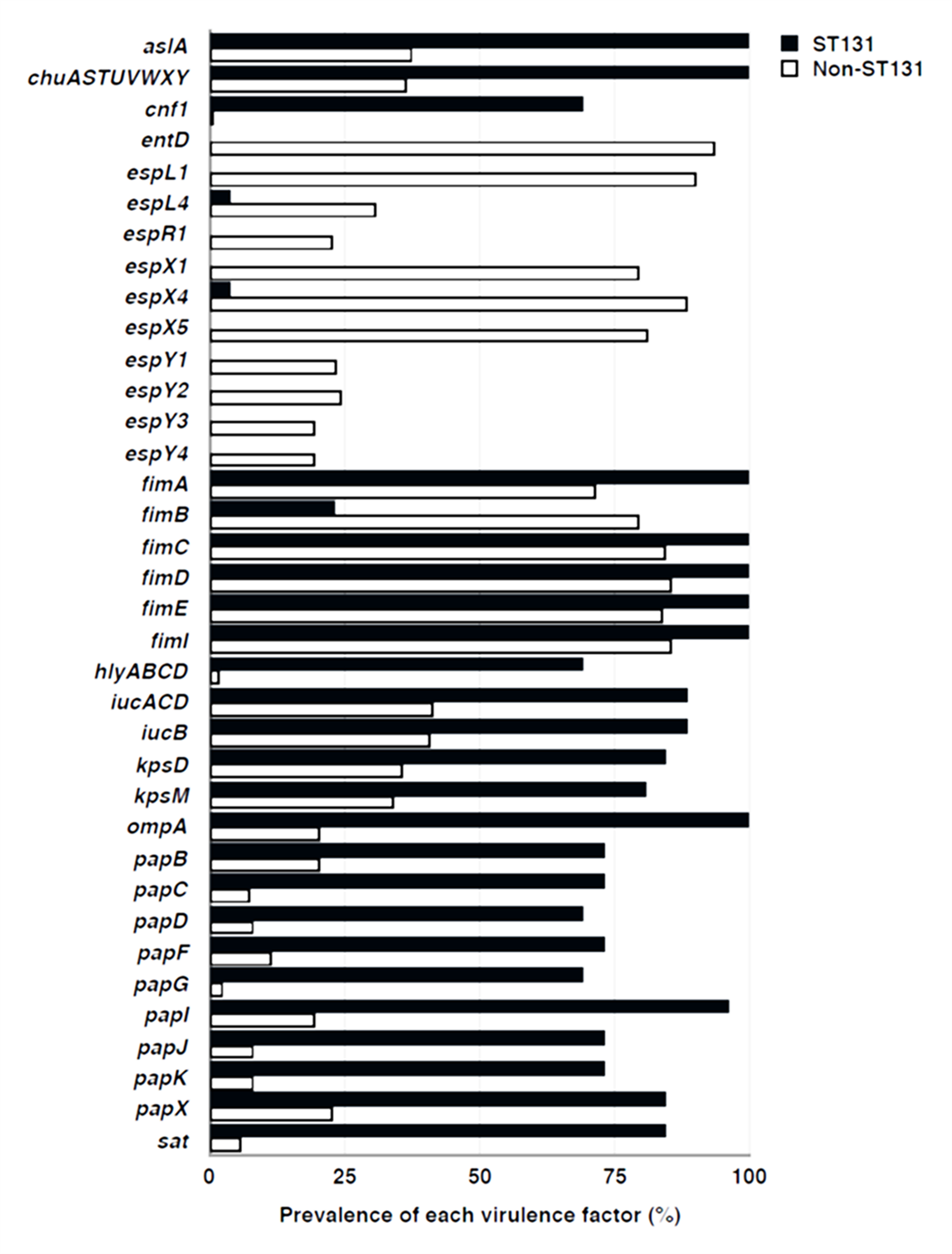


Figure S4 Virulence gene comparison between ST131 and non-ST131 isolates. Graph showing the prevalence of virulence factors in ST131 (n=26) compared to non-ST131 (n=123) in the study isolates. Using Fisher’s exact test at a p-value of 0.05, only those factors over-represented between the two groups are shown.
